# Diagnostic accuracy of BASIC-Q for detection of cognitive impairment in a primary care setting – a cross-validation study

**DOI:** 10.1186/s12877-024-04675-1

**Published:** 2024-01-11

**Authors:** Anne-Britt Oxbøll, Kasper Jørgensen, T. Rune Nielsen, Sofie D. Christiansen, Ann Nielsen, Frans B. Waldorff, Gunhild Waldemar

**Affiliations:** 1 Danish Dementia Research Centre, Department of Neurology, Copenhagen University Hospital- Rigshospitalet, Copenhagen, Denmark; 2 Section of General Practice, Department of Public Health, University of Copenhagen, Copenhagen, Denmark; 3 Department of Clinical Medicine, University of Copenhagen, Copenhagen, Denmark

**Keywords:** Cognitive impairment, Primary health care, Cognitive assessment screening instrument, Test-retest reliability, Validation study

## Abstract

**Objectives:**

This study aims to evaluate the diagnostic accuracy and reliability of a new, brief questionnaire, ‘Brief Assessment of Impaired Cognition– Questionnaire’ (BASIC-Q) for detection of cognitive impairment, primarily developed for use in primary care. BASIC-Q has three components: Self-report, Informant report, and Orientation. Self-report and Orientation are completed by the individual and Informant report is answered by a close relative.

**Methods:**

We included 275 participants ≥ 70 years, without a prior diagnosis of dementia, and with a close relative who agreed to participate as an informant. Participants were included prospectively in 14 general practices in urban and rural Denmark using a convenience sampling method. The Repeatable Battery for the Assessment of Neuropsychological Status (RBANS), the informant-completed Functional Activities Questionnaire (FAQ) and reported memory concern were used as a reference standard for the classification of the participants’ cognitive function.

**Results:**

BASIC-Q demonstrated a fair to good diagnostic accuracy to differentiate between people with cognitive impairment and normal cognition with an area under the ROC curve (AUC) of 0.84 (95% CI 0.79–0.89) and a sensitivity and specificity of 0.80 (95% CI 0.72–0.87) and 0.71 (95% CI 0.63–0.78). A prorated BASIC-Q score derived from BASIC-Q without Informant report had significantly lower classification accuracy than the full BASIC-Q. The test-retest reliability of BASIC-Q was good with an intraclass correlation coefficient of 0.84.

**Conclusion:**

BASIC-Q is a brief, easy-to-use questionnaire for identification of cognitive impairment in older adults. It demonstrated fair to good classification accuracy in a general practice setting and can be a useful case-finding tool when suspecting dementia in primary health care.

**Supplementary Information:**

The online version contains supplementary material available at 10.1186/s12877-024-04675-1.

## Introduction

Dementia is a major global public health challenge, affecting both the individual with dementia and their families and caregivers [1]. Dementia is characterized by cognitive impairment affecting both activities of daily living (ADL) and social functioning [2] and may be caused directly by progressive brain disorders and indirectly by numerous other disease states. The strongest risk factors for dementia are old age and genetic predisposition [3] but in Denmark approximately 35% of the risk may be associated with modifiable risk factors [4, 5]. Early diagnosis allows the patient access to treatment and support and to take active part in decisions about the future [6]. However, the rate of undetected dementia is high, particularly in people with dementia in early stages [7]. The World Health Organization’s (WHO) global action plan 2017–2025 focuses, among other challenges, on undetected dementia and has set as a global target that 50% of people with dementia are diagnosed by 2025 [1]. The modest target of 50% demonstrates that the WHO recognizes the complexity and magnitude of the current underdiagnosis.

In older adults, dementia is often accompanied by physical disorders and psychiatric comorbidities [2] and many people with undiagnosed dementia will therefore often be in close contact with primary care, which mainly consists of general practitioner (GP) and community health care. Hence, community healthcare professionals can be of pivotal importance in the detection of dementia. To our knowledge, no case-finding tools for dementia have been validated in Danish community health care.

This need led to the development of the Brief Assessment of Impaired Cognition (BASIC) and the Brief Assessment of Impaired Cognition Questionnaire (BASIC-Q). BASIC is a short cognitive test to be administered in clinical settings, e.g., GP clinics, and BASIC-Q is designed as a brief questionnaire primarily developed for community healthcare professionals [8, 9].

Two other validated questionnaires developed for detection of cognitive decline, the Cognitive Function Instrument (CFI) [10] and the Informant Questionnaire on Cognitive Decline in the Elderly (IQCODE) [11] are comparable to BASIC-Q. They do, however only have subjective questions on memory, IQCODE for informants only, and CFI for both informants and the individual whereas BASIC-Q also includes objective questions on orientation [8].

BASIC-Q has demonstrated excellent discriminative validity in a memory clinic population in Denmark, and was favorably received by patients and relatives [8]. BASIC-Q requires no extensive training or test materials. Thus, it can be used by healthcare professionals experienced in initiating conversations on health-related topics but who may not be trained to perform cognitive testing.

The aim of the present study was to investigate the diagnostic accuracy and test-retest reliability of BASIC-Q in identifying people with possible cognitive impairment (including mild cognitive impairment (MCI) and dementia) in a sample of patients recruited from primary care.

## Methods

### Participants

Participants were recruited between October 2021 and October 2022. The recruitment took place in GP clinics among patients who consulted their GP for any reason.

Participants were eligible for inclusion if they were ≥ 70 years and had a close relative or friend who agreed to serve as an informant about the participant’s ADL. Subjects were excluded if they had a known diagnosis of dementia, or a condition that judged by the GP would affect the subject’s ability to cooperate in the extended cognitive assessment such as addiction to drugs, alcohol, or medication, severe chronic psychiatric or physical illness or significant sensory deficits. Participants also needed to be sufficiently fluent in Danish to participate in cognitive testing without the assistance of an interpreter.

### Primary health care setting

The participating GP clinics were mainly recruited by one of the authors (FBW). At introduction meetings prior to study start, GPs and the involved staff received information regarding inclusion and exclusion criteria and were trained in the administration of BASIC-Q to ensure it was administered uniformly. The GP clinics received an honorarium for study activities including screening and inclusion of patients.

In total, 14 GP clinics from four out of five administrative regions in Denmark participated in the study. Throughout the study period, two research assistants monitored and provided supervision to the participating clinics.

### BASIC-Q

BASIC-Q is a brief questionnaire with 10 items divided into three components: Self-report, Informant report, and Orientation (Supplementary Table S1).

Self-report contains three questions on subjective perception of memory function. Informant report contains three questions on the cognitive functioning of the individual as perceived by a close relative. Orientation contains three questions on orientation in time and one question regarding the age of the individual. Each question in the Self-report and Informant report has three response categories, with a score range of 0–2 points, ranging from no problems to extensive problems. The orientation questions are scored 0 points for an incorrect response and 2 points for a correct response. The total score ranges from 0 to 20 points with low scores indicating cognitive impairment [8].

Informant report provides valid information, particularly when the patient is cognitively impaired [12]. In situations where reliable informant report cannot be obtained, BASIC-Q may be administered without Informant report and a so-called prorated BASIC-Q score may be used. The conversion table for prorating can be found as supplementary material in a previous publication [8]. In this study, however, Informant report was mandatory for inclusion.

### Study activities

This study was part of a larger study on identification of cognitive impairment in primary care where multiple cognitive tests, and a range of questionnaires were administered.

At the first visit, participants received information about the project, gave informed consent and BASIC-Q was administered. Prior to the administration of BASIC-Q, the GP filled out a form including a brief screening question regarding memory concern: “Does either the patient, a close relative or you yourself have the impression that the patient has problems with his/her memory?” The answer options were ‘yes’ and ‘no’. If the GP checked ‘yes’ the participant would be considered positive for ‘memory concern’. Within two weeks after the first visit, the participants were contacted by a research assistant and a structured telephone interview with questions regarding socio-demographic factors, health, and well-being was administered. To categorize level of education we applied a slightly simplified version of the Danish classification system of educational level, DISCED-15: 1) Primary education, 2) Lower secondary education, 3) Upper secondary education and post-secondary non-tertiary education, 4) Short-cycle tertiary education, 5) Bachelor’s or equivalent level, 6) Master’s, doctoral or equivalent level [13]. The second research visit with extended cognitive assessment with an approximate duration of one and a half hours, was performed by a research assistant within a month after the first visit. The second visit was mainly performed at the GP clinics. However, to accommodate the participants’ needs and physical limitations some visits were performed at the Danish Dementia Research Centre or in the participant’s home.

The extended cognitive assessment included the Mini-Mental State Examination (MMSE) [14], the Repeatable Battery for the Assessment of Neuropsychological Status (RBANS) [15], the Montreal Cognitive Assessment (MoCA) [16], and the Rowland Universal Dementia Assessment Scale (RUDAS) [17] (administered in that order). The 15-item Geriatric Depression Scale (GDS-15) and other questionnaires were also administered. All participants who completed the MMSE and GDS-15 and whose informant completed the Functional Activities Questionnaire (FAQ) [18] were eligible for inclusion. Informants answered the FAQ in a telephone interview or in person if accompanying their relative to the extended cognitive assessment [18].

A subgroup of 60 participants completed BASIC-Q both at the first and second research visit to determine the test-retest reliability of the questionnaire. BASIC-Q was administered by different healthcare professionals responsible for inclusion at the first research visit and by a research assistant at the second visit.

### Classification of participants

**Fig. 1:**
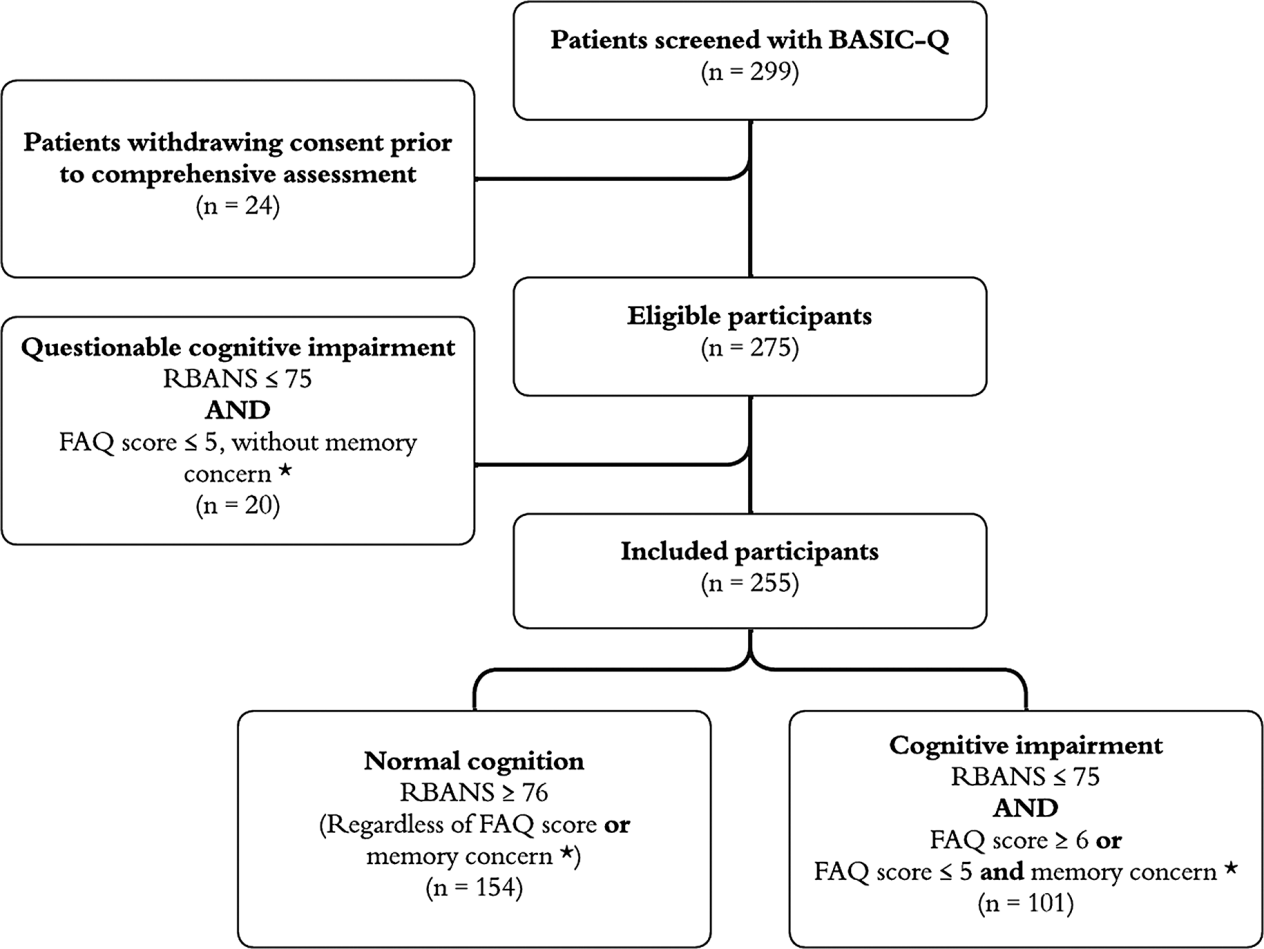
Classification of study participants. * Memory concern: Prior to performing BASIC-Q, the GP registered whether the participant, an informant or GP staff had concerns about the participant's memory function

Based on cognitive performance (RBANS total index score), informant reported independence in everyday instrumental activities (FAQ score), and any concern by the patient, an informant or the GP staff that there had been a decline in the memory functioning of the patient at inclusion, participants were categorized in two main groups: (1) ‘normal cognition’, and (2) ‘cognitive impairment’ (Fig. 1).

The RBANS total index cutoff score was set as 75/76 equivalent to the 5th percentile often used as indicator of impaired performance in clinical settings [19]. Participants with an RBANS total index score ≥ 76 were classified as having ‘normal cognition’. Participants with an RBANS total score ≤ 75, and either a FAQ score ≥ 6, or reported concern regarding memory decline at inclusion, were classified as having ‘cognitive impairment’. RBANS was not administered to participants with an MMSE-score ≤ 20 who were classified as having ‘cognitive impairment’. The FAQ cutoff score of 5/6 was based on a large validation study including participants with MCI and mild Alzheimer’s disease [20].

A group of participants (*n* = 20) could not be classified by the algorithm and were excluded from further analyses. As there was no concern regarding memory decline at inclusion for the participants in this residual group and they were without reported impairment in instrumental ADL, they would generally not be subject to cognitive assessment in a clinical setting.

### Blinding

All study personnel involved in the extended cognitive assessment or classification of participants were blinded to the BASIC-Q results except for the subgroup of participants in whom a BASIC-Q retest was performed.

### Data analysis

Prior to the study, we estimated the base rate of dementia to be approx. 6 to 7% according to previous findings in a Danish GP setting [21]. The required number of participants was calculated as 524 using Buderer’s formula for power estimation [22]. The power estimation was adjusted to 93 during the inclusion period as the proportion of participants with cognitive impairment was substantially higher than expected.

Independent-samples t-tests were conducted to examine differences between groups. Effect sizes were calculated as Hedges *g* [23]. An effect size of 0.2 was considered small, 0.5 was considered medium, and 0.8 was considered large [24]. Receiver operating characteristics (ROC) curves were used to assess the discriminative validity of BASIC-Q applying the described classification algorithm as gold standard. The area under the ROC-curve (AUC) is a general measure of the ability of BASIC-Q to discriminate between subjects with cognitive impairment and normal cognition, and discriminative validity was further determined by calculating sensitivity and specificity. The optimal cutoff score was determined by the Youden index [25].

Negative and positive predictive values (NPV and PPV) at selected base rates of 10%, 25% and 50% were calculated. PPV is the proportion of participants with a positive test result at a given cutoff score who truly have the condition of interest (COI), whereas NPV is the proportion with a negative result truly being without the COI [26].

$${\rm{PPV}}\, = \,{\rm{true}}\,{\rm{positive}}\,{\rm{cases}}\,/\,{\rm{true}}\,{\rm{positive}}\, + \,{\rm{false}}\,{\rm{positive}}\,{\rm{cases}}$$

$${\rm{NPV}}\, = \,{\rm{true}}\,{\rm{negative}}\,{\rm{cases}}\,/\,{\rm{true}}\,{\rm{negative}}\, + \,{\rm{false}}\,{\rm{negative}}\,{\rm{cases}}$$

As indicated by the formula, predictive validity statistics are influenced by the prevalence of the COI. PPV can also be interpreted as an estimate of the probability of the COI for individuals scoring positive at a given cutoff, whereas NPV may work as an estimate of the probability of being without the COI for individuals scoring negative according to the cutoff.

A likelihood ratio (LR) is the ratio of two probabilities [26]. The LR + is the probability of a participant with the COI testing positive, divided by the probability of a participant without the COI testing positive. The LR- is the probability of a participant with the COI testing negative, divided by the probability of a participant without the COI testing negative.

$${\rm{LR}}\, + \, = \,{\rm{sensitivity}}\,/\,\left( {1\, - \,{\rm{specificity}}} \right)$$

$${\rm{LR}}\, - \, = \,\left( {1\, - \,{\rm{sensitivity}}} \right)\,/\,{\rm{specificity}}$$

As indicated by the formula, LRs are independent of the prevalence of the COI. When applied to test scores differing from the optimal cutoff, LRs illustrate the fact that extreme test scores may have greater predictive power than test scores close to the optimal cutoff. LRs associated with BASIC-Q scores above and below the optimal cutoff were calculated to further determine its classification performance.

Two secondary analyses were performed. First, the discriminative validity analyses were repeated using prorated BASIC-Q scores. AUCs were compared using the nonparametric approach by DeLong et al. for correlated ROC curves [27]. Second, discriminative validity was established in a sensitivity analysis in which the participants from the ‘normal cognition’ and ‘cognitive impairment’ groups with a GDS-15 score ≥ 6 were reclassified as a separate group with ‘probable affective disorder’. The GDS-15 cutoff score was based on a Danish validation study [28].

Test-retest reliability was assessed by the intraclass correlation coefficient (ICC) that classifies participants as clusters and the two test results characterized as repeated measures.

All analyses were performed with IBM SPSS statistical software (v28). *P* <.05 (two-tailed) was considered significant.

### Ethical considerations

Written informed consent from all participants, and informed oral consent from all informants, was obtained at time of recruitment. All participants were informed of their rights when participating in a research study. The project was approved by the Danish Data Protection Agency (P-2020-685) and submitted to the Danish Research Ethics Committee that waived the need for ethical approval for the study.

### Patient and public involvement

The aim of this research study was to assess the validity and reliability of BASIC-Q for identifying people with possible cognitive impairment. The outcome measure (gold standard) was determined in advance (by definition) and should not be influenced by patient priorities, experience, and preferences and patients were not involved in the study’s design. However, during the development of the questionnaire, valuable insights were obtained by interviewing patients and caregivers about their attitudes towards BASIC-Q.

The results of the study will be disseminated to healthcare professionals and the public. Each study participant was offered an opportunity to be informed about their own test results.

## Results

### Participants

Two hundred and ninety-nine patients ≥ 70 years were initially recruited and assessed with BASIC-Q in GP clinics. However, later 24 people withdrew consent due to sudden illness or the study being more time consuming than anticipated, and thus 275 participants were eligible for inclusion. Twenty participants with questionable cognitive impairment were excluded and consequently 255 were included in the analyses (Fig. 1). Socio-demographic information is presented in Table 1. Further summary results regarding cognitive performance in four separate age groups are presented in Supplementary Table S2.

**Table 1:** Socio-demographic and cognitive participant characteristics

	Normal cognition	Cognitive impairment	Total
Number	154	101	255
Age (years)	76.5 (5.25)	79.3 (5.10)	77.6 (5.36)
Sex (female/male)	88/66	48/53	136/119
Education (DISCED)*	4.1 (1.34)	3.4 (1.06)	3.8 (1.28)
GDS-15	1 [0–11]	3 [0–13]	2 [0–13]
FAQ	0 [0–19]	6 [0–22]	2 [0–22]
MMSE	28.7 (1.39) [24–30]	24.9 (3.83) [12–30]	27.2 (3.22) [12–30]
RBANS total score	96.7 (13.42) [76–143]	57.9 (11.74) [40–75] **	82.4 (22.71) [40–143]

Age and education are reported as mean and standard deviation. MMSE and RBANS are reported as mean, standard deviation and range. GDS-15 and FAQ are reported as median and range

GDS-15, 15-item Geriatric Depression Scale; FAQ, Functional Activities Questionnaire; MMSE, Mini-Mental State Examination; RBANS, Repeatable Battery for the Assessment of Neuropsychological Status DISCED; Danish International Standard Classification of Education

* Classified according to the classification system used by Statistics Denmark

** RBANS was not performed for participants with an MMSE score < 20 (*n* = 11)

The median time between the first research visit (assessment with BASIC-Q) and the second research visit (extended assessment) was 29.5 days (interquartile range 17–35). The ‘normal cognition’ group (*n* = 154) was significantly younger than the ‘cognitive impairment’ group (*n* = 101) (*t* (253) = -4.21, *p* <.001, *g* = 5.21).

Significant differences with large effect sizes were present between the two groups on BASIC-Q scores (*t* (253) = 9.67, *p* <.001, *g* = 2.97), Self-report (*t* (253) = 5.95, *p* >.001, *g* = 1.36), Orientation (*t* (253) = 5.30, *p* <.001, *g* = 1.34), and Informant report (*t* (253) = 10.31, *p* <.001, *g* = 1.44) (Table 2).

**Table 2:** Performance on BASIC-Q and its components

	Normal cognition (*n* = 154)	Cognitive impairment (*n* = 101)	p-value
BASIC-Q	17.4 (2.33)	13.4 (3.74)	< 0.001
Self-report	4.6 (1.26)	3.5 (1.49)	< 0.001
Orientation	7.9 (0.48)	6.8 (2.04)	< 0.001
Informant report	4.9 (1.35)	3.0 (1.56)	< 0.001

Results are presented as means and standard deviations. Group comparisons were made using independent-samples t-test

### Discriminative validity

**Fig. 2:**
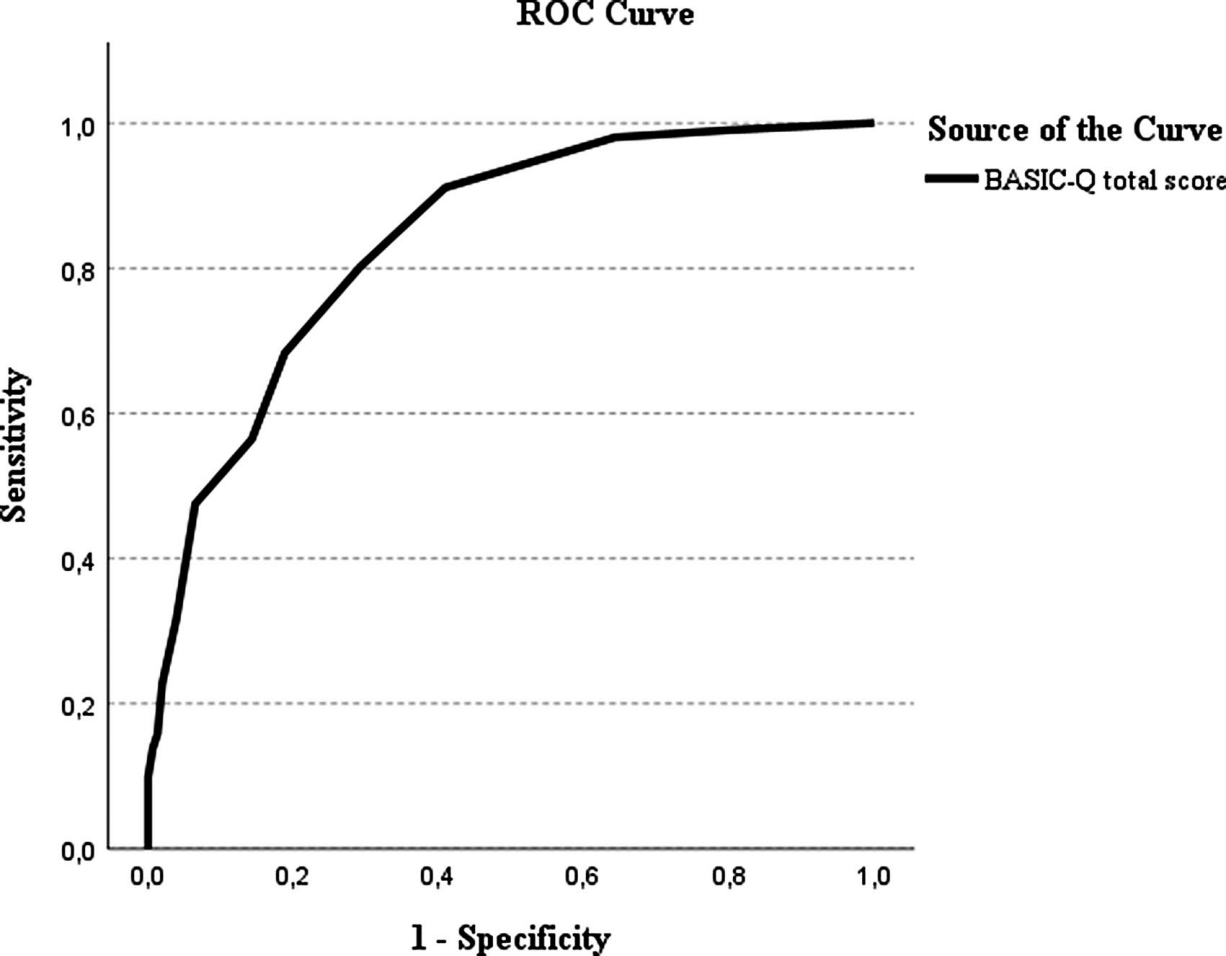
Receiver operating characteristics of BASIC-Q as case-finding tool for cognitive impairment

BASIC-Q had a fair to good accuracy (AUC): 0.84 (95% CI 0.79–0.89) for discriminating between participants with normal cognition and cognitive impairment (Fig. 2).

The optimal cutoff was determined as 16/17, at which BASIC-Q had good sensitivity (0.80) and fair specificity (0.71). At this cutoff the LR + was 2.75, indicating that the probability of a positive result was more than twice as high in participants with cognitive impairment than in participants with normal cognition. The LR- was 0.28 indicating that the probability of a negative result was approximately four times lower in participants with cognitive impairment than in participants with normal cognition (Table 3).

**Table 3:** Classification accuracy of BASIC-Q for cognitive impairment at different cutoff scores

Cutoff	Sensitivity (95% CI)	Specificity (95% CI)	LR+ (95% CI)	LR- (95% CI)
14/15	0.56 (0.47–0.66)	0.86 (0.80–0.91)	3.95 (2.59–6.03)	0.51 (0.40–0.64)
15/16	0.68 (0.59–0.77)	0.81 (0.75–0.87)	3.63 (2.55–5.17)	0.39 (0.29–0.53)
16/17*	0.80 (0.72–0.87)	0.71 (0.63–0.78)	2.75 (2.11–3.58)	0.28 (0.19–0.42)
17/18	0.91 (0.85–0.96)	0.59 (0.51–0.67)	2.23 (1.82–2.72)	0.15 (0.08–0.29)
18/19	0.98 (0.94-1.00)	0.36 (0.28–0.44)	1.53 (1.35–1.72)	0.06 (0.01–0.22)

Abbreviations: CI, confidence interval; LR+, Positive likelihood ratio; LR–, Negative likelihood ratio

*Optimal cutoff score

At a base rate of cognitive impairment of 0.40 in the present sample we found a high NPV (0.85, 95% CI 0.78–0.90), but a modest PPV (0.64, 95% CI 0.56–0.72). Further PPV and NPV estimates for base rates of cognitive impairment set at 10%, 25% and 50% are presented in Table 4.

**Table 4:** Predictive validity estimates at different cutoff scores and base rates of cognitive impairment

	Base rate 10%		Base rate 25%		Base rate 50%	
Cutoff	**PPV (95% CI)**	**NPV (95% CI)**	**PPV (95% CI)**	**NPV (95% CI)**	**PPV (95% CI)**	**NPV (95% CI)**
14/15	0.30 (0.18–0.44)	0.95 (0.91–0.97)	0.57 (0.45–0.69)	0.85 (0.80–0.90)	0.80 (0.71–0.87)	0.66 (0.59–0.73)
15/16	0.28 (0.18–0.40)	0.96 (0.93–0.98)	0.55 (0.44–0.66)	0.89 (0.83–0.93)	0.78 (0.70–0.85)	0.72 (0.64–0.85)
16/17*	0.23 (0.15–0.33)	0.97 (0.94–0.99)	0.48 (0.38–0.57)	0.91 (0.86–0.95)	0.73 (0.66–0.80)	0.78 (0.70–0.85)
17/18	0.20 (0.13–0.28)	0.99 (0.96-1.00)	0.43 (0.35–0.51)	0.95 (0.90–0.98)	0.69 (0.62–0.76)	0.87 (0.79–0.93)
18/19	0.15 (0.10–0.20)	0.97 (0.91–0.99)	0.34 (0.27–0.41)	0.99 (0.94-1.00)	0.60 (0.54–0.67)	0.94 (0.85–0.98)

Abbreviations: CI, confidence interval; PPV, Positive predictive validity; NPV, negative predictive validity

* Optimal cutoff score

For the estimates at a cutoff at 16/17 with a base rate at 10% we found a very low PPV (0.23; 95% CI 0.15–0.33), which may suggest that BASIC-Q would be ineffective as a screening tool as the probability of an individual with a positive test result is truly cognitively impaired is less than 0.25 at this base rate.

However, applying BASIC-Q in a targeted way with a pre-test probability of approx. 50% (equivalent to a base rate of 50%), both the PPV and NPV would be fair.

### Prorated BASIC-Q

**Fig. 3:**
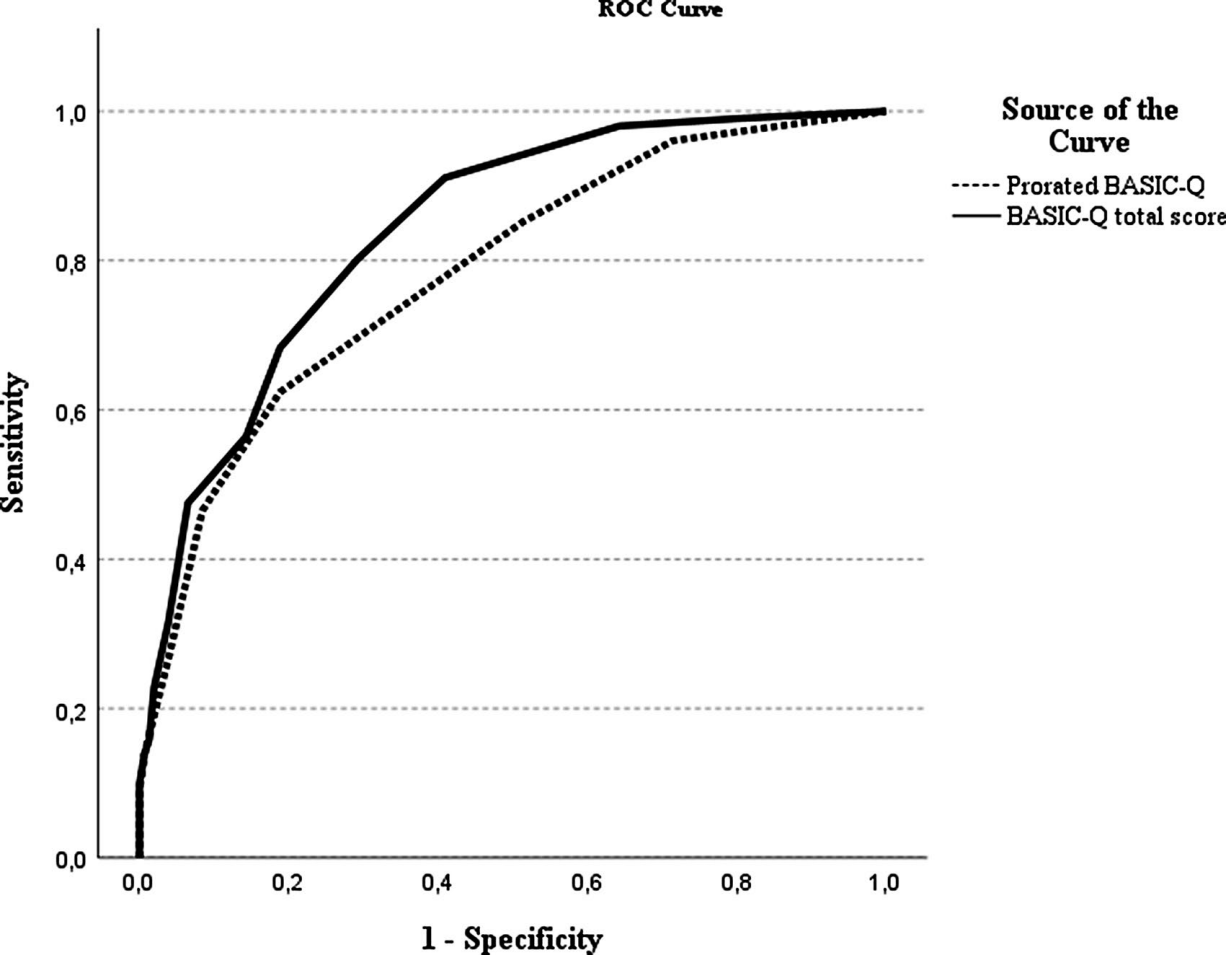
Receiver operating characteristics of prorated BASIC-Q as case-finding tool for cognitive impairment

The discriminative analysis was repeated using the prorated BASIC-Q score. The prorated BASIC-Q score had fair classification accuracy for normal cognition versus cognitive impairment (AUC 0.78; 95% CI 0.73–0.84) (Fig. 3).

At the optimal cutoff 16/18 (a prorated BASIC-Q score of 17 cannot be obtained), specificity was good (0.81), but sensitivity was poor (0.62) (Supplementary Table S3). Compared to the full BASIC-Q, the prorated BASIC-Q has a significantly lower classification accuracy (*z* = 3.59, *p* =.000).

### ‘Probable affective disorder’ classified separately

**Fig. 4:**
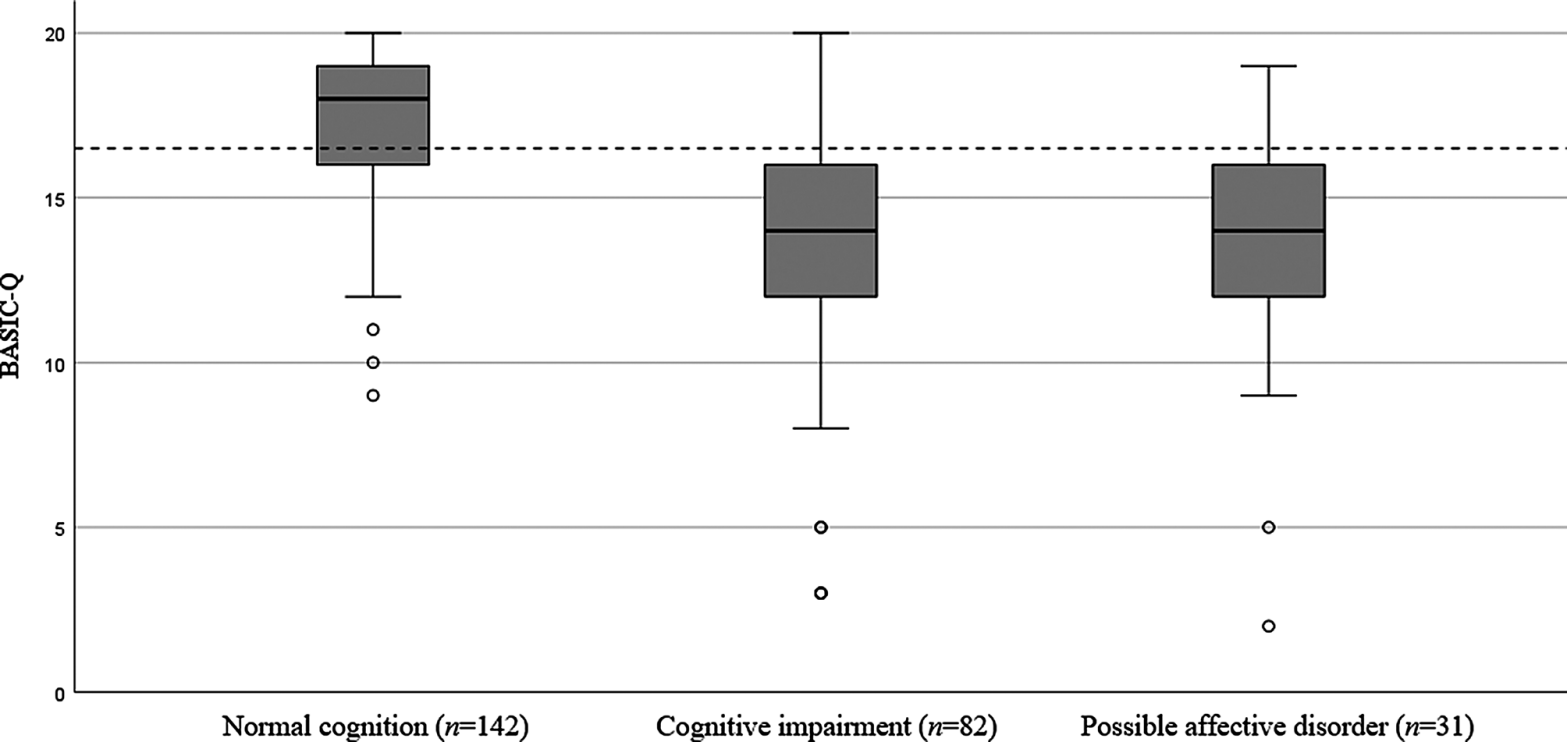
Boxplot of BASIC-Q distribution across three participant groups. The horizontal reference line represents the optimal cutoff score (16/17) for differentiating between the ‘normal cognition’ and ‘cognitive impairment’ group

The discriminative validity analysis was repeated after reclassifying participants with a GDS-15 score ≥ 6 from the ‘normal cognition’ and ‘cognitive impairment’ groups as a separate group, ‘probable affective disorder’ (*n* = 31), regardless of their cognitive performance. This did not change the classification accuracy (AUC 0.83; 95% CI 0.78–0.89) (Supplementary Figure S1), sensitivity (0.78), or specificity (0.73) considerably. BASIC-Q performances of the three groups are illustrated in Fig. 4.

### Test-retest reliability

BASIC-Q had good test-retest reliability with an ICC of 0.84. The test-retest reliability was good for the components Orientation and Informant report (ICC 0.84 and 0.83, respectively) and moderate for Self-report (ICC 0.72).

## Discussion

BASIC-Q was primarily developed as a dementia case-finding tool for use in primary care settings. It is brief and well suited for healthcare professionals working in a primary care setting without specialized expertise in dementia or experience with cognitive testing.

This study is the first to validate BASIC-Q in a primary care setting. In accordance with the primary validation study [9] we found the optimal cutoff to be 16/17. At this cutoff, we found a good sensitivity (0.80) and fair specificity (0.71), poor PPV (0.64) but good NPV (0.85).

We previously validated BASIC-Q in a memory clinic setting and found a substantially higher discriminative validity with excellent sensitivity (0.92) and specificity (0.97) and excellent classification accuracy (AUC = 0.98) [8]. The difference in the discriminative validity between primary care and the memory clinic setting may be explained by sampling differences. Patients referred to memory clinics are typically highly selected, whereas a GP clinic sample is far more heterogeneous. BASIC-Q is primarily developed for use in primary care for the primary purpose of deciding whether to motivate the individual for further evaluation by the GP. In this study, presence of cognitive symptoms was not a requirement for participation, however, some GPs recruited patients for whom they had concern regarding their cognitive status. The base rate of cognitive impairment (40%) in our study may seem high but is not markedly different from the combined base rates of MCI (35.3%) and dementia (14.6%) found in a large, population-based, Norwegian study [29]. The sensitivity and specificity of BASIC-Q in a primary care setting may potentially increase if the questionnaire was applied in a targeted way based on concern from patients, relatives, caregivers, or healthcare professionals rather than used as a screening tool. However, further research is needed to determine this.

Two comparable questionnaires, the IQCODE [11], and the CFI [10], for detection of cognitive impairment have shown a similar discriminative validity. A systematic review on the validity of the IQCODE found that IQCODE had sensitivities in the range of 0.65 to 0.96 and specificities between 0.69 and 0.94 in various study samples [30]. In a sample comparable to the sample in this study, the sensitivity and specificity was found to be respectively 0.73 and 0.76 for the self-rated CFI and 0.84 and 0.84 for the proxy-rated CFI [31]. The classification accuracy of BASIC-Q therefore seems comparable to similar tools when applied in a similar population.

Patients with both cognitive and depressive symptoms represent a particular challenge. As cognitive decline may influence the validity of the GDS-15, there is a possibility of incorrect classification of affective disorder. In this study, BASIC-Q could not distinguish between individuals with probable affective disorder and cognitive impairment. After reclassifying participants with a GDS-15 score ≥ 6 from the ‘normal cognition’ and ‘cognitive impairment’ groups into the ‘probable affective disorder’ group, BASIC-Q showed a good AUC (AUC 0.83; 95% CI 0.78–0.89), fair sensitivity (0.78), and specificity (0.73). Regardless of whether poor BASIC-Q performance is caused by cognitive or affective symptoms, the BASIC-Q result can prompt evaluation at a GP who will assess the individual more thoroughly.

Compared with the full BASIC-Q, the prorated BASIC-Q had significantly poorer classification accuracy (AUC of 0.78 vs. 0.84) and sensitivity (0.62 vs. 0.80). The poor sensitivity indicates that almost 40% of the participants with cognitive impairment were not identified by the prorated BASIC-Q. The specificity of the prorated BASIC-Q was good (0.81), and a negative prorated BASIC-Q result may help exclude cognitive impairment. People with cognitive impairment or dementia often have a lack of insight in their cognitive status [32]. Previous studies have shown that self-reported cognitive symptoms can be unreliable in individuals with dementia even in the early stages. Therefore informants are an important source for information [8, 31]. As the prorated BASIC-Q only consists of self-perceived memory and four orientation questions, the low sensitivity mostly likely reflects that the instrument is highly influenced by the Self-report when answered by a person with a lack of insight. Therefore, it could be problematic to apply BASIC-Q to these respondents. In persons with a lack of insight and a limited social network, cognitive impairment could be difficult to detect, unless the healthcare professionals can provide information about changes in cognition in the individual. The prorated BASIC-Q should therefore only be used in situations where an informant is not available as it is less valid than the full BASIC-Q.

BASIC-Q had a good reliability (ICC 0.84). As BASIC-Q was administered first by GP staff, and later by the research assistant at the second visit, the results reflect a combination of interrater and test-retest reliability. This is similar to common practice in a primary care setting where BASIC-Q will often be administered and readministered by different healthcare professionals (e.g., when the first assessment is inconclusive, and BASIC-Q is readministered at a later date). The reliability of the individual components Informant report (ICC 0.83) and Orientation (ICC 0.84) was also good. The reliability for the Self-report component was a little lower (ICC 0.72) which may reflect difficulties in self-monitoring for individuals with cognitive impairment.

A strength of our study is that the study population was recruited from primary health care, which BASIC-Q is mainly intended for. Further, the study population resembled the background population in people > 70 years in Denmark with more women than men and the GP clinics represented geographically urban and rural areas of Denmark. Both medical doctors and nurses in the GP clinics administered BASIC-Q, reflecting usual clinical practice.

Our study also had some limitations. For practical reasons, participants in this study did not undergo a full clinical diagnostic evaluation at a memory clinic. Instead, a combination of RBANS, FAQ and memory concern was applied to classify the participants. Previous studies of RBANS have shown a good association between RBANS scores and Alzheimer’s disease biomarkers and also that RBANS can discriminate between individuals with Alzheimer’s disease and cognitively intact individuals [33–35]. Secondly, our sample is not fully representative of a typical GP population aged 70 + years as we excluded patients who, according to the judgement of the GP, would not be able to complete the extended cognitive assessment. Therefore, our sample may be healthier and better functioning than the typical GP population. A third possible limitation is the exclusion of a subgroup of patients (*n* = 20) who could not be classified according to our algorithm in neither the ‘normal cognition’ nor ‘cognitive impairment’ group. Fourthly, the informants who answered questions about the participants’ memory were identified by the participants themselves and we have no knowledge about the cognitive status of the informants. Primary healthcare professionals face a similar challenge when using BASIC-Q in clinical settings.

Ineffective communication can result in delayed treatment or misdiagnosis. Providing a standardized tool could serve to increase the level of awareness of signs of cognitive impairment among healthcare professionals. BASIC-Q provides a quantitative measure that can be easily interpreted and serve as a basis for communication about cognitive symptoms. It can contribute to a common “language” among healthcare professionals. BASIC-Q may help make case-finding more systematic in securing a uniform manner of detecting cognitive impairment in community settings and BASIC-Q may also contribute to the decision-making process for healthcare professionals in recommending further evaluation at the GP clinic.

In conclusion, BASIC-Q had fair to good classification accuracy for identifying people in a community health care with cognitive impairment. We found that the full BASIC-Q has a significantly higher accuracy than prorated BASIC-Q and it should be a priority to obtain responses from informants. BASIC-Q is brief and easy to administer, does not require specialized training and is therefore a useful tool when suspecting cognitive impairment in primary care. BASIC-Q is not a diagnostic instrument, but rather a case-finding tool, and may be used to qualify primary healthcare professionals’ decisions about whether to recommend individuals to consult their GP for further evaluation.

### Electronic supplementary material

Below is the link to the electronic supplementary material.

**Supplementary Material 1: Table S1.** Brief Assessment of Impaired Cognition Questionnaire (BASIC-Q)

**Supplementary Material 2: Table S3.** Classification accuracy of BASIC-Q and prorated BASIC-Q for cognitive impairment at different cutoff scores

**Supplementary Material 3: Table S2.** RBANS, BASIC-Q and MMSE performance for separate age groups

**Supplementary Material 4: Figure S1.** Receiver operating characteristics of BASIC-Q as case-finding tool for cognitive impairment – ‘probable affective disorder’ separated from the main groups

## Data Availability

The data that support the findings of this study are available upon reasonable request from the corresponding author, AO.

## Abbreviations

BASIC: Brief Assessment of Impaired Cognition
BASIC-Q: Brief Assessment of Impaired Cognition Questionnaire
GP: General practitioner
CFI: Cognitive Function Instrument
IQCODE: Informant Questionnaire on Cognitive Decline in the Elderly
MMSE: Mini-Mental State Examination
RBANS: Repeatable Battery for the Assessment of Neuropsychological Status
MoCA: Montreal Cognitive Assessment
RUDAS: Rowland Universal Dementia Assessment Scale
GDS-15: 15-item Geriatric Depression Scale
FAQ: Functional Activities Questionnaire
NPV: Negative predictive value (NPV and PPV)
PPV: Positive predictive value
COI: Condition of interest
LR–: Negative likelihood ratio
LR +: Positive likelihood ratio
ROC: Receiver operating characteristics
AUC: Area under the ROC-curve
ICC: Intraclass correlation coefficient
MCI: Mild cognitive impairment
