# Supplementary material for: Diagnostic accuracy of BASIC-Q for detection of cognitive impairment in a primary care setting – a cross-validation study
Source: BMC Geriatr. 2024 Jan 11;24:53. doi: 10.1186/s12877-024-04675-1 (PMC10785536; doi:10.1186/s12877-024-04675-1)
Supplement: Supplementary file 1 — Supplementary Material 1: Table S1. Brief Assessment of Impaired Cognition Questionnaire (BASIC-Q) [file 12877_2024_4675_MOESM1_ESM.docx]

**Supplementary Table S1 Brief Assessment of Impaired Cognition Questionnaire (BASIC-Q)**

| **Component** | **Description** | **Score range** |
| --- | --- | --- |
| 1. Self-report | ·         Compared to previously, do you feel that your memory has declined substantially? | 0-6 |
|  | ·         Do you need more help from others to remember appointments, family occasions, or holidays? |  |
|  | ·         Do you have more trouble recalling names, finding the right words, or completing sentences? |  |
|  |  |  |
|  | Scoring: To a great extent = 0 points; To some extent = 1 point; No = 2 points. |  |
|  |  |  |
| 2. Orientation | What is the year? | 0-8 |
|  | What is the month? |  |
|  | What day of the week is it? |  |
|  | How old are you? |  |
|  |  |  |
|  | Scoring: Correct answer = 2 points; Wrong answer = 0 points |  |
|  |  |  |
| 3. Informant report | Compared with a few years ago, how is your spouse / parent / family member / this person at: | 0-6 |
|  | ·         Remembering things that have happened recently? |  |
|  | ·         Recalling conversations a few days later? |  |
|  | ·         Remembering what day and month it is? |  |
|  |  |  |
|  | Scoring: Much worse = 0 points; A bit worse = 1 point; Unchanged = 2 points. |  |
|  |  |  |
| BASIC-Q total score | | 0-20 |

The optimal cutoff score for cognitive impairment is 16/17. A low score indicates cognitive impairment.
