# Supplementary material for: Diagnostic accuracy of BASIC-Q for detection of cognitive impairment in a primary care setting – a cross-validation study
Source: BMC Geriatr. 2024 Jan 11;24:53. doi: 10.1186/s12877-024-04675-1 (PMC10785536; doi:10.1186/s12877-024-04675-1)
Supplement: Supplementary file 2 — Supplementary Material 2: Table S3. Classification accuracy of BASIC-Q and prorated BASIC-Q for cognitive impairment at different cutoff scores [file 12877_2024_4675_MOESM2_ESM.docx]

**Supplementary Table S3** Classification accuracy of BASIC-Q and prorated BASIC-Q for cognitive impairment at different cutoff scores

|  | Cutoff | Sensitivity (95% CI) | Specificity (95% CI) |
| --- | --- | --- | --- |
| BASIC-Q | 15/16 | 0.68 (0.59-0.77) | 0.81 (0.75-0.87) |
|  | 16/17* | 0.80 (0.72-0.87) | 0.71 (0.63-0.78) |
|  | 17/18 | 0.91 (0.85-0.96) | 0.59 (0.51-0.67) |
| Prorated BASIC-Q | 14/16 | 0.47 (0.37-0.56) | 0.92 (0.87-0.95) |
|  | 16/18* | 0.62 (0.53-0.71) | 0.81 (0.75-0.87) |
|  | 18/20 | 0.85 (0.77-0.91) | 0.49 (0.41-0.57) |

* Optimal cutoff score

Abbreviations: CI, confidence interval
