# Supplementary material for: Diagnostic accuracy of BASIC-Q for detection of cognitive impairment in a primary care setting – a cross-validation study
Source: BMC Geriatr. 2024 Jan 11;24:53. doi: 10.1186/s12877-024-04675-1 (PMC10785536; doi:10.1186/s12877-024-04675-1)
Supplement: Supplementary file 3 — Supplementary Material 3: Table S2. RBANS, BASIC-Q and MMSE performance for separate age groups [file 12877_2024_4675_MOESM3_ESM.docx]

**Supplementary Table S2 RBANS, BASIC-Q and MMSE performance for separate age groups**

| Age group | RBANS total score | BASIC-Q | MMSE |
| --- | --- | --- | --- |
| 70-74 y (*n*=84) | 91.3 (23.19) | 16.8 (2.98) | 28.2 (2.66) |
| 75-79 y (*n*=90) | 78.6 (20.74) | 15.4 (4.02) | 26.8 (3.33) |
| 80-84 y (*n*=51) | 75.6 (22.68) | 15.6 (3.11) | 26.9 (2.78) |
| 85+ y (*n*=30) | 79.3 (19.46) | 14.7 (3.79) | 26.0 (4.24) |
| Total (*n*=255) | 82.4 (22.71) | 15.8 (3.56) | 27.2 (3.22) |

RBANS, Repeatable Battery for the Assessment of Neuropsychological Status; BASIC-Q, Brief Assessment of Impaired Cognition Questionnaire (BASIC-Q); MMSE, Mini‐Mental State Examination.
