# Supplementary material for: Diagnostic accuracy of BASIC-Q for detection of cognitive impairment in a primary care setting – a cross-validation study
Source: BMC Geriatr. 2024 Jan 11;24:53. doi: 10.1186/s12877-024-04675-1 (PMC10785536; doi:10.1186/s12877-024-04675-1)
Supplement: Supplementary file 4 — Supplementary Material 4: Figure S1. Receiver operating characteristics of BASIC-Q as case-finding tool for cognitive impairment – ‘probable affective disorder’ separated from the main groups [file 12877_2024_4675_MOESM4_ESM.docx]

**Supplementary Figure S1** Receiver operating characteristics of BASIC-Q as case-finding tool for cognitive impairment – ‘probable affective disorder’ separated from the main groups


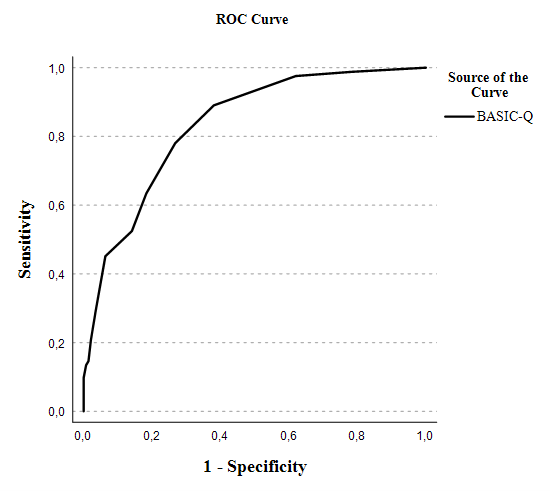


Normal cognition (*n*=142), Cognitive impairment (*n*=82)

Area under the ROC curve (AUC): 0.83 (95% CI 0.78-0.89)

Abbreviation: CI, confidence interval.
